# The Anti-Leukemic Activity of Natural Compounds

**DOI:** 10.3390/molecules26092709

**Published:** 2021-05-05

**Authors:** Coralia Cotoraci, Alina Ciceu, Alciona Sasu, Eftimie Miutescu, Anca Hermenean

**Affiliations:** 1 Department of Hematology, Faculty of Medicine, Vasile Goldis Western University of Arad, Rebreanu 86, 310414 Arad, Romania; sasu.alconia@uvvg.ro; 2 “Aurel Ardelean” Institute of Life Sciences, Vasile Godis Western University of Arad, Rebreanu 86, 310414 Arad, Romania; alinaciceu80@gmail.com (A.C.); hermenean.anca@uvvg.ro (A.H.); 3 Department of Gastroenterology, Faculty of Medicine, Vasile Goldis Western University of Arad, Rebreanu 86, 310414 Arad, Romania; miutescu.eftimie@uvvg.ro; 4 Department of Histology, Faculty of Medicine, Vasile Goldis Western University of Arad, Rebreanu 86, 310414 Arad, Romania

**Keywords:** antioxidants, flavonoids, anti-leukemic, myeloid leukemia, lymphoblastic leukemia

## Abstract

The use of biologically active compounds has become a realistic option for the treatment of malignant tumors due to their cost-effectiveness and safety. In this review, we aimed to highlight the main natural biocompounds that target leukemic cells, assessed by in vitro and in vivo experiments or clinical studies, in order to explore their therapeutic potential in the treatment of leukemia: acute myeloid leukemia (AML), chronic myeloid leukemia (CML), acute lymphocytic leukemia (ALL), and chronic lymphocytic leukemia (CLL). It provides a basis for researchers and hematologists in improving basic and clinical research on the development of new alternative therapies in the fight against leukemia, a harmful hematological cancer and the leading cause of death among patients.

## 1. Introduction

Cancer is one of the leading causes of death worldwide and a major challenge for the public health system [1]. The incidence of cancer is constantly increasing and is estimated to increase by 70% over the next 20 years [2].

Conventional anticancer therapies have limited efficacy and are associated with many side effects, such as hepatotoxicity, myelosuppression, or tumor lysis syndrome [3]. Chemotherapy and radiation therapy are frequently correlated with side effects, such as hair loss, loss of appetite, diarrhea, vomiting, liver damage, and neurological disorders [4]. Therefore, it is necessary to find new therapeutic approaches with high efficacy and fewer side effects. The main treatments used in leukemia are radiotherapy, hyperthermia, and chemotherapy. Conventional drug treatment is associated with cytotoxicity and systemic side effects. Therefore, efforts in cancer treatment are focused on finding strategies that can specifically target tumor cells without affecting normal cells [5]. Understanding the molecular mechanisms involved in hematologic cancers is useful in developing of the new therapeutic strategies that target various molecular abnormalities. Recently, there has been an increase in molecularly targeted therapies approved by the FDA in various types of leukemia, but there are insufficient data on the use of these drugs. Thus, in the case of AML, several agents are available for various clinical stages, but the best response rates were obtained by combining new molecularly-targeted treatments with conventional induction chemotherapy [6]. However, the patients experience short-term nausea/vomiting, diarrhea, hair loss, mouth sores, infection, rash; and for the long-term, organ dysfunction, chemobrain, fatigue, neuropathy, as well as resistance of leukemia cells to chemotherapy drugs [7,8,9], highlighting the need for the development of less toxic and targeted therapies.

Recent advances in understanding carcinogenesis have led to the synthesis of new drugs that target specific receptors [10]. The development of new antitumor agents is an important strategy in the fight against cancer [11]. The development of new anticancer agents derived from natural sources is currently being pursued [12]. Secondary metabolites from plants, such as flavonoids, alkaloids, terpenoids, saponins, and others, are important sources of anticancer agents [13,14,15]. Different types of herbal formulations, such as flavonoids and various enzymes, play an important role in cancer by preventing DNA damage and increasing the level of antioxidants in the body with lower side effects [16]. Lately, many phytochemicals isolated from different parts of the plant have been tested by in vitro and in vivo experiments to find biological effects against different diseases, such as cancer.

Over 60% of anti-tumor drugs that have shown high efficacy in clinical use have been obtained from plants, aquatic organisms, and microorganisms. The anticancer effect of these natural products is mediated by various mechanisms, as apoptosis, modulation of the immune system, and inhibition of angiogenesis [17].

There are several plant-derived compounds used in the treatment of hematologic cancers. The vinca alkaloids, vincristine and vinblastine, the first US FDA-approved anticancer agents in plants, are used to treat lymphomas, including Hodgkin’s disease and acute lymphoblastic leukemias in combination with chemotherapy [18,19]. Etoposides, a compound used in the treatment of various types of leukemias and lymphomas, and teniposides used in various types of hematological cancers, either alone or in combination with chemotherapeutic drugs, are semi-synthetic plant derivatives [20,21].

Cancer chemoprevention is a new approach to cancer management. This therapeutic strategy uses non-cytotoxic drugs and natural agents to inhibit carcinogenesis [22] and block progression to invasive cancer [10]. Secondary metabolites in plants, enzymes, and other compounds play an important role in combating various types of cancer [23]. Chemoprevention includes DNA damage protection, which initiates the process of neoplastic transformation or can reverse the progression of preinvasive lesions. The effectiveness of this approach has been highlighted by epidemiological observations, in experimental models of animal carcinogenesis, knock-out models, tumor cell lines, and clinical studies [10].

In this review, we aimed to highlight the main biologically active compounds which target leukemic cells, assessed by in vitro and in vivo experiments or clinical studies, in order to explore their therapeutic potential in treatment of leukemia.

The biologically active compounds with antileukemic activity presented in the below tables are of plant origin and they are widespread in the plant kingdom. For example: luteolin is a flavone found in carrots, celery, peppers, cabbage, broccoli, onion leaves, apple skins, parsley, basil, thyme, and mint [24,25]; quercetin is found in many fruits and vegetables such as apples, cherries, berries, onions, asparagus, and red leaf lettuce [26]; apigenin is contained in *Artemisia* [27], *Achillea* [28,29], *Matricaria* [30], and *Tanacetum* [31] genera; epigallocatechin-gallate (EGCG) is the main constituent of green tea [32]; curcumin is a phenolic compound found in the rhizomes of *Curcuma longa* L., commonly known as turmeric [33]; thymoquinone is a monoterpene isolated from *Nigella sativa* seeds [34]. It is also found in high concentration in the *Monarda fistulosa* plant, also known as wild bergamot [35]; emodin is a natural anthraquinone derivative [36] extracted from various plants, such as *Rheum officinale* and *Polygonam cuspidatum* [37]; parthenolide is a sesquiterpene lactone extracted from the leaves of the medicinal plant *Tanacetum parthenium* [38].

This wide range of natural compounds with anti-leukemic potential provides a basis for researchers and hematologists in improving basic and clinical research on the development of new alternative therapies in the fight against leukemia.

## 2. Natural Compounds in Acute Myeloid Leukemia (AML)

Acute myeloid leukemia (AML) is the most common type of acute leukemia among adults [5]. This is an aggressive hematological malignancy characterized by an extremely proliferative accumulation of immature and dysfunctional myeloid cells [39] which infiltrates bone marrow, blood, and other tissues [40]. Additionally, leukemic cells show an increase proliferative capacity and altered hematopoietic differentiation [41].

Although most patients with AML experienced partial remission after conventional treatment, such as chemotherapy, they face a number of problems, such as the risk of recurrence, malignant cell resistance, and side effects that diminish the therapeutic value of these treatments [42]. Recurrence is common and the chances of survival are lower for a long term in most cases [43].

The main difficulty in the treatment of AML is chemoresistance, and CD34 + AML cells indicate poor prognosis and resistance to spontaneous apoptosis [44]. The emergence of multidrug resistance (MDR) in chemotherapeutic agents is an important obstacle in the treatment of AML. The discovery of new therapeutic agents that can be used to overcome MDR is becoming a challenge in clinical practice [37].

To date, polyphenols having cytotoxic effect on AML cells were identified [45]. Deng et al. (2017) demonstrated that luteolin extracted from *Reseda odorata L*., inhibited the growth of leukemic cell lines by inducing apoptosis through blocking of the RSK1 pathway, as well as by inhibiting their ability to migrate [46]. Other studies demonstrated a selective inhibitory activity against Fms-like tyrosine kinase 3 (FLT3), a highly expressed tyrosine kinase receptor in patients with AML and induced a strong cytotoxic effect in MV4-11 leukemic cells [47].

Quercetin has been shown to have an antitumor effect in various experimental models using tumor cell lines, including AML [48,49,50]. The antitumor activity of quercetin has been correlated with its ability to inhibit proliferation and induced cell death in AML cells [48,51]. Quercetin induced AML cell apoptosis through Fas-mediated extrinsic pathways [51] and mitochondrial-derived intrinsic pathways [48]. It also had antitumor effect in acute T-cell lymphoblastic leukemia (ALL) and chronic myeloid leukemia (CML) [52,53].

Delphinidin showed antiproliferative effects against human acute promyelocytic leukemia (APL) NB4 cell line, a subtype of acute myeloid leukemia. Delphinidin had a cytotoxic effect on NB4 cells, induced activation of caspase-8 and -9 and -3 and decreased Bid expression and mitochondrial membrane potential (ΔΨm). Delphinidine-induced cytotoxicity was more pronounced in NB4 cells compared to normal peripheral blood mononuclear cells (PBMNCs) [54].

Genistein has been shown to have antiproliferative activity on tumor cells, being an alternative therapy for the treatment of patients with AML [55].

Parthenolide induced specific toxicity to leukemic cells and leukemic stem cells (LSCs) without causing damage to normal hematopoietic cells [56]. Parthenolide has been shown to be effective in inducing specific apoptosis to LSCs in AML. Due to poor bioavailability, the antileukemic activity of parthenolide has not been demonstrated in vivo [57,58]. In order to increase water solubility, parthenolide analogs have been developed [59] that showed high bioavailability and bioactivity in vivo [57]. The chemically modified parthenolide analog, dimethylamino-parthenolide, showed an oral bioavailability of ~70% compared to intravenous administration in experimental models performed in mice and dogs and an improvement in the selective eradication of AML and of their progenitor stem cells [57].

Martínez-Castillo et al. (2018) studied the effects of curcumin in two cell lines derived from chronic and acute myeloid leukemia, respectively, HL-60 and K562 cells. K562 cells showed a higher sensitivity to cytostatic and cytotoxic effects of curcumin compared to HL-60 cells. Curcumin induced G1 phase blockade in HL-60 cells and G2/M phase blockade in K562 cells. Curcumin induced apoptosis in cell lines derived from chronic and acute myeloid leukemia by distinct cellular mechanisms. Thus, curcumin-induced apoptosis in HL-60 cells was caspase-dependent, whereas in K562 cells, they underwent apoptosis in a caspase-independent manner [60].

Boswellic acid acetate, a 1:1 mixture of α-boswellic acid acetate and β-boswellic acid acetate, isolated from *Boswellia carterri*, showed cytotoxic effects against six myeloid leukemia cell lines. This cytotoxic effect was mediated by the induction of apoptosis. Over 50% of cells underwent apoptosis after treatment with 20 mg/mL boswellic acid acetate for 24 h [61].

The main pharmacological effects exerted by natural compounds against acute myeloid leukemia (AML) are summarized in Table 1.

**Figure 1:**
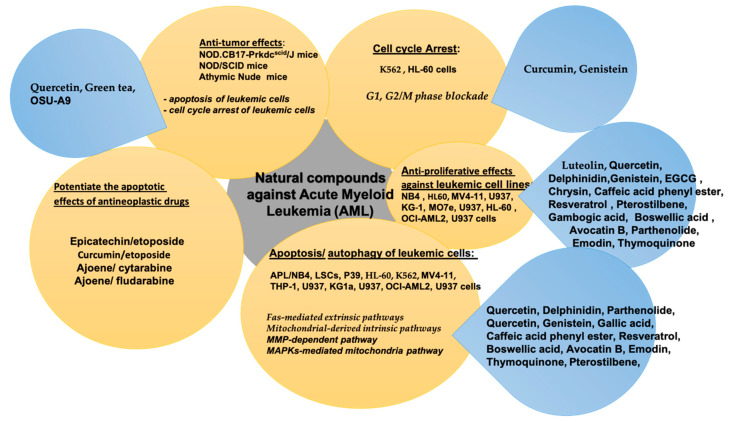
Natural compounds against acute myeloid leukemia (AML).

The natural compounds with anti-tumoral activity against acute mieloid leukemia (AML) by in vitro and in vivo experiments or synergic activity with antineoplastig drugs, are summarized in Figure 1.

## 3. Natural Compounds in Chronic Myeloid Leukemia (CML)

Chronic myeloid leukemia (CML), BCR-ABL1-positive, also known as chronic myelogenous leukemia, is defined as a myeloproliferative neoplasm consisting predominantly of proliferating granulocytes [83]. This has an incidence of 1–2 cases per 100,000 adults [84]. Approximately 95% of patients with CML have t (9; 22) translocation (q34; q11.2) [85]. CML affects both peripheral blood and bone marrow [83].

Fusion of the Abelson gene (ABL1) on chromosome 9 with the cluster breakpoint region (BCR) on chromosome 22 generates the oncoprotein BCR-ABL, an active tyrosine kinase that induces cytokine-independent cell proliferation, which causes excessive accumulation of myeloid cells in hematopoietic tissues [86]. The Bcr-Abl oncoprotein activates several downstream pathways, responsible for inducing cell proliferation, loss of adhesion, cell differentiation blocking, and inhibition apoptosis [87,88].

The main pharmacological effects exerted by natural compounds against chronic myeloid leukemia (CML) are summarized in Table 2.

**Figure 2:**
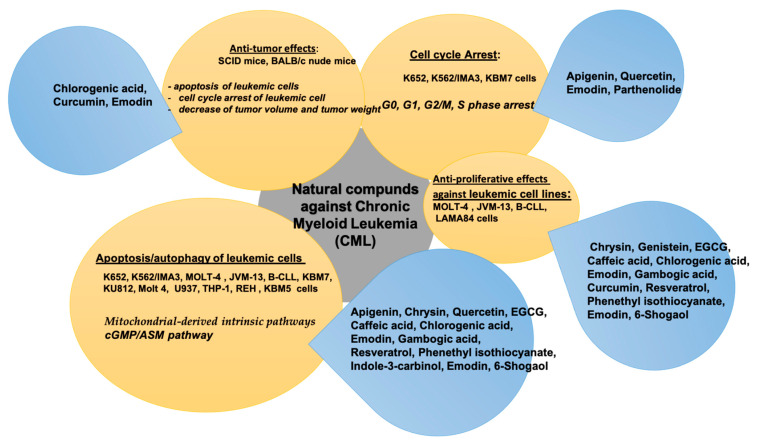
Natural compounds against chronic myeloid leukemia (CML).

The natural compounds with anti-tumoral activity against chronic myeloid leukemia (AML) by in vitro and in vivo experiments, are summarized in Figure 2.

## 4. Natural Compounds in Acute Lymphoblastic Leukemia (ALL)

Acute T-cell lymphoblastic leukemia (T-ALL) is an aggressive malignant blood disorder [112]. Currently, the T-ALL treatment protocols include high doses of chemotherapeutics, which have significant toxic side effects [113,114]. Natural products with various biological activities and specific selectivity have served as important sources of antitumor agents that have been developed for clinical use [115].

Anthocyanins, a subclass of flavonoids, are glycosides of anthocyanidins [116]. Blueberries are an important source of anthocyanins [117]. Anthocyanins showed, anti-mutagenesis and anti-carcinogenesis activity [118,119]. They have been shown to have a strong antitumor effect by inducing a pro-apoptotic mitochondrial-mediated response [120].

Anthocyanins from blueberry extract (Antho 50) induced apoptosis in Jurkat cells by decreasing the expression of Polycomb group proteins. This effect was mediated by an increase in intracellular ROS and depolarization of the mitochondrial membrane [117]. In another study, two anthocyanins extracted from blackcurrant juice, delphinidin-3-*O*-glucoside and delphinidin-3-*O*-rutinoside, induced apoptosis in human Jurkat leukemic cells [121]. Additionally, blackcurrant juice and blackcurrant extract inhibited proliferation, induced cell cycle arrest in the G2/M phase, and apoptosis in Jurkat cells. These effects have been associated with increased expression of p73 and caspase 3, Akt and Bad dephosphorylation, and down-regulation of UHRF1 and Bcl-2 [121].

The main pharmacological effects exerted by natural compounds against acute lymphoblastic leukemia (ALL) are summarized in Table 3.

**Figure 3:**
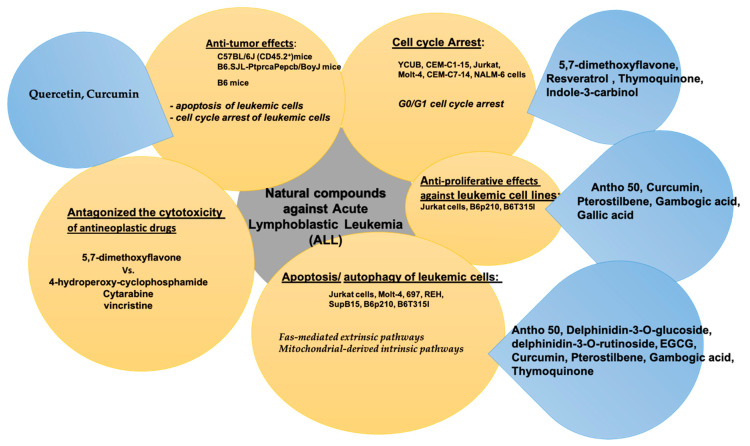
Natural compounds against acute lymphoblastic leukemia (ALL).

The natural compounds with anti-tumoral activity against acute lymphoblastic leukemia (ALL) by in vitro and in vivo experiments or antagonizing activity against cytotoxicity of antineoplastic drugs, are summarized in Figure 3.

## 5. Natural Compounds in Chronic Lymphocytic Leukemia (CLL)

Chronic lymphocytic leukemia (CLL) is the most common type of hematologic cancer in the western countries (22–30%) [134,135]. CLL is a monoclonal lymphoproliferative disorder characterized by the proliferation and accumulation of morphologically mature, but immunologically dysfunctional B-cell lymphocytes [136]. CLL B cells interact with their microenvironment, and B cell survival is enhanced by contact with bone marrow stromal cells. Therefore, the lifespan of B cells increases, causing their abnormal accumulation [137]. The main sites of the disease include peripheral blood, spleen, lymph nodes, and bone marrow [136]. It mainly affects adults [138].

Although there are many therapeutic protocols, CLL is still an incurable disease [138]. Current treatment options include conventional chemotherapy, monoclonal antibodies, and hematopoietic transplantation [139]. These standard treatment methods are not sufficient to eliminate all CLL cells and have a number of side effects. Additionally, standard treatment promotes the development of resistance to treatment and most treated patients relapsed. Therefore, it is necessary to develop new therapeutic strategies that could eliminate apoptosis-resistant CLL cells. Recently, there has been a growing interest in the use of agents derived from natural compounds for cancer therapy [140].

Bcl-2 plays a key role in regulating cellular responses to treatment due to its pro- and anti-apoptotic properties [141]. The anti-apoptotic protein Bcl-2 is overexpressed in several hematological malignancies, including CLL. This overexpression is considered to be responsible for defective apoptosis in CLL [142].

The effects of polyphenols on cell proliferation, gene regulation, and apoptosis have been studied on several cancer cell lines [143].

Alhosin et al. (2015) demonstrated that a standardized blueberry extract containing 50% anthocyanins (Antho 50) had the ability to induce apoptosis in CLL B cells via the Bcl-2/Bad pathway. They evaluated the pro-apoptotic effect of Antho 50 on CLL B cells from 30 patients and on peripheral blood mononuclear cells (PBMCs) from healthy subjects. The main phenolic compounds in cranberry extract responsible for the pro-apoptotic effect in CLL B cells were delphinidin-3-*O*-glucoside and delphinidin-3-*O*-rutinoside. Antho 50-induced apoptosis has been associated with caspase-3 activation, down-regulation of UHRF1, dephosphorylation of Akt and Bad, and down-regulation of Bcl-2 [144].

Luteolin significantly induced apoptosis in chronic lymphocytic leukemia (CLL) cell lines by increasing caspase activity and triggering the intrinsic apoptotic pathway [145].

The main pharmacological effects exerted by natural compounds against chronic lymphocytic leukemia (CLL) are summarized in Table 4.

**Figure 4:**
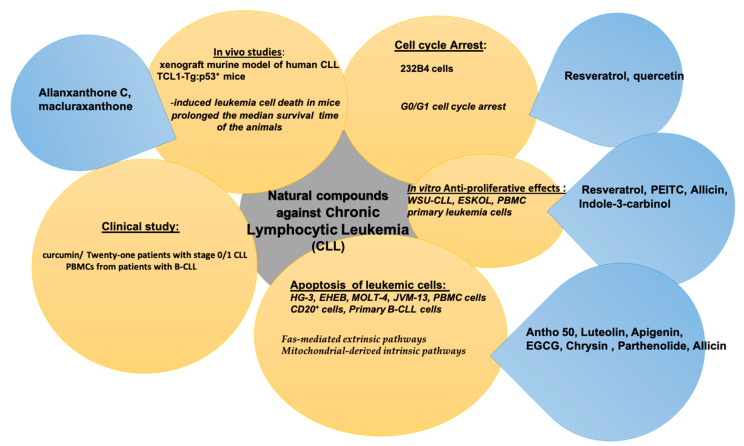
Natural compounds against chronic lymphocytic leukemia (CLL).

The natural compounds with anti-tumoral activity against chronic lymphocytic leukemia (CLL) by in vitro and in vivo experiments or antagonizing activity against cytotoxicity of antineoplastic drugs, are summarized in Figure 4.

## 6. Clinical Trials and Synergic Activity with Conventional Anti-Leukemic Drugs

Several clinical studies are published in database ClinicalTrials.Gov regarding the anti-tumor action of biactive compounds and synergies with anti-neoplastic therapy of leukemias.

The effect of genistein was tested in a phase I/II clinical study in combination with decitabine in pediatric relapsed refractory malignancies. Genistein was administered orally twice daily from day 2 to day 21, followed by a 7-day break (clinical trial number: NCT02499861). The aim of the research includes assessment of a tolerated dose of the combination of intravenous decitabine with oral genistein for children with refractory or recurrent solid malignancies and leukemia. The adverse events of the combination therapy and clinical benefit in phase IIa of the study measured by either volumetric MRI for solid tumor or by bone marrow aspiration or biopsy for leukemia at the end of cycles 2, 4, 6, 9, and 12 were assessed. To date, the results are not yet published in the database ClinicalTrials.Gov.

The efficacy of concomitant administration of curcumin and colecalciferol was investigated in a phase II trial in the treatment of patients with chronic lymphocytic leukemia in stage 0-II, previously untreated and small lymphocytic lymphoma (clinical trial number: NCT02100423).

Given that green tea extract contains ingredients that can slow the growth of certain cancers, its effect was tested in a phase I/II trial in the treatment of patients with chronic lymphocytic leukemia in stage 0, I, or II (clinical trial number: NCT00262743). In the phase I trial, patients were given orally 400 to 2000 mg of green tea extract (Polyphenon E) twice a day for 6 months [158]. In the phase II trial, oral administration of 2000 mg of Polyphenon E twice daily for 6 months was well tolerated [159]. Most patients experienced a decrease in absolute lymphocyte count (LAC) and lymphadenopathy following treatment with Polyphenon E [158,159].

## 7. Conclusions

In this review, we presented the natural compounds that have shown an anti-leukemic activity in experimental studies on different cell lines or primary cultures, preclinical and clinical studies, results that could propose them in subsequent therapeutic protocols of different types of leukemia: acute myeloid leukemia (AML), chronic myeloid leukemia (CML), acute lymphocytic leukemia (ALL), and chronic lymphocytic leukemia (CLL). Mechanistically, they demonstrated the ability to induce cell cycle blockage and apoptosis or autophagy in cancer cells, as well as inhibition of proliferation/migration and tumor progression, antagonizing activity of cytotoxicity exerted by antineoplastic drugs, or exerted synergy with conventional therapy. Although in vitro results are promising, most bioactive compounds have not yet been tested in preclinical or clinical studies. Moreover, some of the compounds are not soluble and therefore have a reduced bioavailability when administered orally (e.g., flavonoids), which reduces their potential. Therefore, special formulations or chemical modification are needed to increase the bioactive potential. Overall, nature provides a wide range of bioactive compounds with anti-leukemic potential, and extensive research is still needed for them to be considered viable therapeutic options for the treatment of various types of leukemia.

## Figures and Tables

**Table 1:** Pharmacological effects of natural compounds in acute myeloid leukemia (AML).

Bioactive Compound	In Vitro/In Vivo/Clinical Study	Cancer Cell Line and Animal Model	Bioactive Effect	References
Luteolin	In vitro	MOLM-13 and Kasumi-1 cells	-inhibited leukemic cell proliferation and induced apoptosis by inhibition of the RSK1 pathways -triggered RSK-dependent antileukemic responses with dephosphorylation of Bad or KIBRA	[46]
EGCG	In vitro	NB4 and HL60 cells	-induced cell death in myeloid leukemic cells -↑ DAPK2 levels in AML cells -EGCG/ATRA cotreatment of myeloid leukemic cells enhanced neutrophil differentiation	[62]
(−)-Epicatechin	In vivo	Brown Norway rats	↑ the in vivo apoptotic effect of etoposide ↑ the oxidative stress induced by etoposide in leukemic rats	[63]
Quercetin	In vitro	MV4-11 and HL-60 cells	-promoted AML cell death -induced caspase-dependent apoptosis in AML cells -induced apoptosis via mitochondrial pathway -suppressed VEGFR2 and PI3K/Akt signaling pathway	[39]
Quercetin	In vitro	HL60 and U937 cells	-down-regulated DNMTs and STAT3 -induced H3 and H4 global acetylation -enriched H3ac and H4ac in the promoter region of the apoptosis pathway genes and increased their transcription levels ↓ the protein expression of class I HDACs in leukemia cells -caused proteasome-mediated protein degradation of HADCs in leukemia cells -down-regulated DNMTs and HADCs at the protein levels, in xenograft models	[64]
Quercetin	In vitro	human myeloid leukemia KG-1 cells	-cytotoxicity effect against KG-1 cells -augmented the TRAIL-induced cell death in KG-1 cells ↑ mRNA expression levels of DR genes in acute myeloid KG-1 cells ↓ mRNA expression of apoptosis inhibitor genes in the acute myeloid KG-1 cells ↓ mRNA expression of NF-κB (p65 subunit) gene in the acute myeloid KG-1 cells	[5]
Quercetin	In vitro	P39 cells	-induced apoptosis in P39 leukemia cells ↓ Bcl-2, Bcl-xL, Mcl-1 down-regulation ↓ Bax -induced mitochondrial translocation, triggering cytochrome c release and caspases activation	[65]
	In vivo	NOD.CB17-Prkdc^scid^/J mice	-induced the expression of FasL protein ↑ cell arrest in G1 phase of the cell cycle ↓ in CDK2, CDK6, cyclin D, cyclin E, and cyclin A proteins ↓ Rb phosphorylation ↑ p21 and p27 expression -induced autophagosome formation in P39 cell line ↓ tumor volume in P39 xenografts in vivo	
Quercetin and green tea	In vivo	NOD/SCID mice	↓ tumor growth in HL-60 xenografts accompanied by decreased expression of anti-apoptotic proteins, Bcl-2, BCL-xL, and Mcl-1 and increased expression of Bax, a pro-apoptotic protein -induced apoptosis of leukemic cells -induced activation of caspase-3 -induced cell cycle arrest of leukemic cells -mediated G1 phase cell cycle arrest in HL-60 xenografts -induced conversion of LC3-I to LC3-II ↑ autophagy in leukemic cells	[41]
Chrysin	In vitro	MO7e cells	-inhibited SCF/c-Kit complex-induced cell proliferation in human myeloid leukemia cells -inhibited SCF-induced phosphorylation of c-Kit -inhibited cell proliferation in MO7e cells by blocking c-Kit phosphorylation	[66]
Genistein	In vitro	MV4-11 and HL-60 cells	-arrested the mTOR pathway leading to down-regulation of protein synthesis -induced cell death via apoptosis -regulatory effects on the cell cycle of the two cell lines, with the induction of G2⁄M phase arrest in HL-60 cells but not in MV4-11 cells	[67]
Gallic acid	In vitro	THP-1 and MV411 cells	-induced caspase-dependent apoptosis of AML cell lines, primary MNC and CD34 stem/progenitors isolated from AML patients via caspase-dependent pathway -enhanced cytarabine and daunorubicin efficacy in vitro cell culture system and in vivo xenograft model -inhibited mitochondrial respiration in AML cells, leading to decreased ATP production and oxidative stress -acted on AML cells via Akt/mTOR-dependent inhibition of mitochondrial respiration	[68]
Caffeic acid phenyl ester (CAPE)	In vitro	U937 cells	↓ cell viability of U937 cells -induced the mitochondria-mediated apoptosis-release of cytochrome C, reduction of Bcl-2 expression, increase of Bax expression, activation/cleavage of caspase-3, and activation/cleavage of PARP	[69]
Curcumin	In vitro	HL-60 cells	-potentiated the cytotoxic effect of etoposide -intensified apoptosis and phosphorylation of the histone H2AX induced by this cytostatic drug in leukemic HL-60 cells -curcumin modified the cytotoxic action of etoposide in HL-60 cells through intensification of ROS production -enhanced the antileukemic activity of etoposide in BNML rats and induced apoptosis of BNML cells more efficiently than etoposide alone, but this treatment protected nonleukemic B-cells from apoptosis	[70]
	In vivo	Brown Norway rats with acute myeloid leukemia (BNML)		
Resveratrol	In vitro	CD34^+^ CD38^−^ KG1a cells	↓ pLKB1 in CD34^+^ CD38^−^ KG1a cells ↑ the expression of SIRT1 in CD34^+^ CD38^−^ KG1a cells -induced senescence and apoptosis of CD34^+^CD38^−^ KG1a cells	[71]
Resveratrol	In vitro	HL-60 cells	↓ CSC-related Shh expression, Gli-1 nuclear translocation, and cell viability in IL-6-treated HL-60 cells -had synergistic effect with Shh inhibitor cyclopamine on inhibiting cell growth	[72]
Resveratrol	In vitro	U937 and MV-4-11 cells	-interacted synergistically with HDACIs in human myeloid leukemia cells -coadministration with HDACIs led to enhanced DNA damage, mitochondrial injury, and caspase-3, caspase-9, and caspase-8 activation -blocked HDACI-mediated RelA acetylation and NF-κB activation -induced S-phase accumulation and sensitized leukemia cells to HDACIs	[73]
Pterostilbene	In vitro	MV4-11 HL-60, U937, and THP-1 AML cells	-suppressed cell proliferation in various AML cell lines -induced G0/G1-phase arrest when expressions of cyclin D3 and CDK2/6 were inhibited -induced cell apoptosis occurred through activation of caspases-8/-9/-3, and a MMP-dependent pathway -treatment of HL-60 cells with PTER induced sustained activation of ERK1/2 and JNK1/2, and inhibition of both MAPKs by their specific inhibitors significantly abolished the PTER-induced activation of caspases-8/-9/-3 -PTER-induced cell growth inhibition was only partially reversed by the caspase-3-specific inhibitor, Z-DEVE-FMK -promoted disruption of LMP and release activated cathepsin B -induced HL-60 cell death via MAPKs-mediated mitochondria apoptosis pathway	[74]
Gambogic acid	In vitro	U937 and HL-60 cells	-had cytotoxic effect on AML cells -inhibited cell growth and promoted differentiation in U937 and HL-60 cells ↑ the expression of p21waf1/cip1 in the two cell lines	[75]
3-*O*-acetyl-11-keto-β-boswellic acid (AKBA)	In vitro	HL-60 cells	-inhibited dose-dependent proliferation of HL-60 and apoptosis rate of HL-60 cells -changed the cell cycle by increasing of G(1) phase and decreasing of S phase -anti-proliferation and apoptosis-inducing effects on HL-60 cells	[76]
Boswellic acid acetate	In vitro	NB4, SKNO-1, nK562, U937, ML-1, and HL-60 cells	-inhibited cell growth and induced cell toxicity of myeloid leukemia cell lines -induced apoptosis through a p53-independent pathway by activation of caspase-8 induced proteolysis of Bid ↓ mitochondrial membrane potential without production of hydrogen peroxide ↑ the levels of DR4 and DR5 mRNA in apoptotic cells	[61]
Avocatin B	In vitro	OCI-AML2 cells	↓ human primary AML cell viability without effect on normal peripheral blood stem cells -selectively toxic toward leukemia progenitor and stem cells -induced mitochondria-mediated apoptosis -inhibited fatty acid oxidation and ↓ NADPH levels, resulting in ROS-dependent leukemia cell death	[77]
Parthenolide	In vitro	U937 cells	-inhibited growth of U937 cells -induced apoptosis in U937 cells ↓ the CD38+ population of U937 cells ↓ osteopontin gene expression in U937 cells	[78]
Parthenolide	In vitro	AML cells, bcCML cells, normal bone marrow, and umbilical cord blood cells	-induced apoptosis in primary human AML cells and bcCML cells sparing normal hematopoietic cells -targeted preferentially leukemic but not normal progenitor and stem cell activity	[43]
	In vivo	Nonobese diabetic/severe NOD/SCID mice	-the molecular mechanism of PTL mediated apoptosis is associated with inhibition of NF-κB, proapoptotic activation of p53, and increased ROS -the activity of PTL triggers LSC-specific apoptosis	
Emodin	In vitro	AML HL-60/ADR cells	-induced growth inhibition and apoptotic effects in resistant HL-60/ADR cells in vitro as well as in the HL-60/H3 xenograft models in vivo ↑ chemosensitivity of AML cells to Ara-C, inhibited leukemic cell growth, and improved survival in mouse xenograft model of AML	[37]
	In vivo	BALB/C-nude mice		
Emodin	In vitro	NB4, MR2 and primary AML cells	-inhibited cell proliferation in NB4 cells, MR2 cells, and primary AML cells -enhanced differentiation induction of ATRA in retinoid-responsive NB4 cells as well as in retinoid-resistant MR2 cells -induced cell apoptosis in NB4 cells, MR2 cells, and primary AML cells -the apoptotic induction in AML cells was associated with the activation of caspase cascades involving caspase-9, caspase-3, and PARP cleavage -induced the activation of the caspase-dependent pathway -induced the degradation of RARα protein in NB4 and MR2 cells -inhibited activation of the PI3K/Akt signaling pathway in AML cells -inhibited p-Akt at Ser473 as efficiently as mTOR at Ser2448 -suppressed the phosphoration of mTOR downstream targets, 4E-BP1 and p70S6K	[79]
Thymoquinone	In vitro	HL-60 cells	↓ HL-60 cell viability -induced apoptosis in HL-60 cells ↓ the expression of WT1 and BCL2 genes	[80]
Ajoene	In vitro	KG1 cells	↓ bcl-2-expression ↑ the inhibitory effect of the two chemotherapeutic drugs, cytarabine and fludarabine, on Bcl-2-expression in KGI cells -the two drugs, cytarabine and fludarabine, ↑ the activated caspase-3 level in KGI myeloid leukemia cells -ajoene enhanced the activation of caspase-3 in both cytarabine- and fludarabine-treated KGI cells	[81]
OSU-A9	In vitro	HL-60 and THP-1 cells and primary leukemia cells from AML patients	-induced cytotoxicity in AML cell lines and primary leukemia cells from AML patients ↓ cyclin A and cyclin B1 in AML cell lines -induced apoptosis, caspase activation, and PARP cleavage in AML cell lines -induced autophagy but not autophagic cell death in AML cell lines -OSU-A9-mediated cytotoxicity and hypophosphorylation of Akt were dependent on the generation of ROS -suppressed the growth of THP-1 xenograft tumors and prolonged the survival of tumor-bearing athymic nude mice	[82]
	In vivo	athymic nude mice		

**Legend:** ↑ increased/up-regulated; ↓ decreased/down-regulated; RSK1—ribosomal S6 kinase 1; RSK (ribosomal S6 kinase); Bad—Bcl-2-associated death promoter; KIBRA—kidney/brain protein; EGCG—epigallocatechin-3-gallate; HL-60—human promyelocytic leukemia; DAPK2—death-associated protein kinase 2; 67LR—67 kDa laminin receptor; ATRA—all-trans retinoic acid; VEGFR2—vascular endothelial growth factor receptor 2; PI3K/Akt signaling pathway—phosphatidylinositol 3-kinase/protein kinase B signaling pathway; DNMTs—DNA methyl transferases; STAT3—signal transducer and activator of transcription 3; HDACs—histone deacetylases; TRAIL—apoptosis-inducing ligand; mRNA—messenger ribonucleic acid; NF-κB—nuclear factor-κB; Bcl-2—B-cell lymphoma-2; Bcl-xL—B-cell lymphoma-extra-large; Mcl-1—myeloid cell leukemia 1; Bax—Bcl-2-associated X protein; CDK2—cyclin-dependent kinase 2; CDK6—cyclin-dependent kinase 6; Rb—retinoblastoma protein; SCF—stem cell factor; mTOR—mechanistic target of rapamycin; MNC—mononuclear cells; ATP—adenosine triphosphate; Akt—protein kinase B; CAPE—caffeic acid phenyl ester; PARP—poly(ADP-ribose) polymerase; BNML—Brown Norway rats with acute myeloid leukemia; pLKB1—phosphorylated liver kinase B1; SIRT1—Sirtuin 1; IL-6—interleukin 6; CSC—cancer stem cell; Shh—sonic hedgehog; Gli-1—glioma-associated oncogene homolog 1; HDACIs—histone deacetylase inhibitors; PTER—pterostilbene; MMP—mitochondrial membrane permeabilization; ERK1/2—extracellular signal-regulated kinase 1/2; JNK1/2—c-Jun N-terminal protein kinase 1/2; MAPKs—mitogen-activated protein kinases; Z-VAD-FMK—carbobenzoxy-valyl-alanyl-aspartyl-[O-methyl]-fluoromethylketone; LMP—lysosomal membrane permeabilization; AKBA—3-*O*-acetyl-11-keto-β-boswellic acid; DR4 and DR5—death receptors 4 and 5; NADPH—nicotinamide adenine dinucleotide phosphate; ROS—reactive oxygen species; PTL—parthenolide; bcCML—blast crisis CML; LSCs—leukemia stem cells; Ara-C—cytarabine; RARα—retinoic acid receptor α; p-Akt—Akt phosphoration; WT1—Wilms’ tumor 1 gene.

**Table 2:** Pharmacological effects of natural compounds in chronic myeloid leukemia (CML).

Bioactive Compound	In Vitro/In Vivo/Clinical Study	Cancer Cell Line and Animal Model	Bioactive Effect	References
Apigenin	In vitro	K652 and K562/IMA3 cells	-induced cytotoxic and apoptotic effects in K562 and K562/IMA3 cells -induced loss of mitochondrial membrane potential in both K562 and K562/IMA3 cells ↑ caspase-3 activity in both K562 and K562/IMA3 Cells -arrested cell cycle progression in G2/M phase in K562 cells -induced S phase arrest in K562/IMA3 cells -regulated a set of genes in K652 and K562/IMA3 cells	[89]
Chrysin	In vitro	MOLT-4 and JVM-13 cell lines, B-CLL cells derived from 28 patients and PBMC from 16 healthy subjects	↓ the viability of of leukemic cells -induced apoptosis of peripheral blood lymphocytes isolated from human CLL patients via mitochondrial pathway -induced the activation of proapoptotic Bax ↓ the expression of antiapoptotic Bcl-2 protein -released cytochrome c from mitochondria into cytosol -activated caspase-3, subsequently leading to the activation of apoptosis of B-CLL cells	[90]
Quercetin	In vitro	K-562 cells	-induced apoptosis in K-562 cells -abrogated K-562 cells proliferation ↓ genes expression of HSP70, Bcl-X(L), and FOXM1 -improved Bax, caspase-3, and caspase-8 expression	[91]
Quercetin	In vitro	KBM7 cells	-inhibited KBM7 cell proliferation -induced cell apoptosis -blocked cell cycle at G1 phase ↓ the mRNA and protein expression of Smoothened and Glioma1 (Gli1) ↓ Bcl-2 and cyclin D1 ↑ p53 and caspase-3 expression -inhibited Hh signaling and its downstream targets in the KBM7 cells	[92]
Quercetin and curcumin		K562 cells	-induced changes in several genes in 10 different pathways related to cell proliferation, apoptosis, cell cycle, inflammation, hypoxia, and oxidative stress ↓ CDKN1B, AKT1, IFN-γ ↑ BTG2, CDKN1A, FAS	[93]
Genistein	In vitro	CML and CFU-Mix BFU-E and CFU-GM hematopoietic progenitors	-suppressed colony formation -suppressed progenitor cell growth ↓ marrow BCR/ABL+ progenitors -exerted a strong antiproliferative effect on CFU-Mix, BFU-E, and CFU-GM ↓ the percentage of leukemic LTC-IC -induced apoptosis of CML mononuclear and CD34^+^	[94]
EGCG	In vitro	K562, K562R, KCL-22, BaF3/p210 and BaF3/p210^T315I^ cell lines	-inhibited the proliferation of CML cell lines and primary CML cells ↓ the mitochondrial membrane permeability of CML cell lines -induced the apoptosis of CML cells through caspase-independent and AIF-mediated cell death pathways -suppressed the expression of Bcr/Abl and phospho-Bcr/Abl in CML cell -regulated Bcr/Abl downstream JAK2/STAT3/AKT and p38-MAPK/JNK signaling pathways in CML	[95]
EGCG	In vitro	KU812 cells	-induced ASM activation and lipid raft clustering in CML cells -induced phosphorylation of protein kinase Cδ at Ser664 -induced cell death via the cGMP/ASM pathway in CML cells	[96]
Caffeic acid	In vitro	K562 cells	-induced mitochondrial membrane depolarization, genomic DNA fragmentation, and phosphatidylserine exposure, hallmarks of apoptosis ↓ cell proliferation -↑ expression of two cell cycle repressor genes, CDKN1A and CHES1	[97]
Chlorogenic acid	In vitro	K562, Molt 4, U937, THP-1, REH cell lines	-induced apoptosis of several Bcr-Abl–positive CML cell lines and primary cells from CML patients in vitro -destroyed Bcr-Abl–positive K562 cells in vivo -no effect on the growth and viability of Bcr-Abl–negative lymphocytic and myeloid cell lines and primary CML cells -↓ viability of Bcr-Abl–positive cells in vitro and in vivo -induced apoptosis of Bcr-Abl–positive cells -inhibited autophosphorylation of p210Bcr-Abl fusion protein -modulated MAP kinase pathways in K562 cells	[98]
	In vivo	Nude female mice		
Emodin	In vitro	K562 cells	-inhibited the growth of K562 cells harboring BCR-ABL in vitro and in vivo -induced apoptosis by inhibition of PETN/PI3K/Akt level and deletion of BCR-ABL	[99]
Gambogic acid	In vitro	K562 cells	-inhibited the viability of K562 cells -induced the accumulation of autophagic vacuoles and up-regulation of two autophagy-related proteins (Beclin 1 and LC3) ↓ mRNA levels of BCR/ABL fusion genes and SQSTM1/sequestosome 1 (p62) protein levels -induced cell death through autophagy and apoptosis pathways in CML K562 cells	[100]
Gambogic acid	In vitro	KBM5, KBM5-T315I, and K562 cells	-induced apoptosis and cell proliferation inhibition in CML cells -induced caspase activation in CML cells -inhibited the proteasome function in CML cells -down-regulated Bcr-Abl protein and inhibited its downstream signaling -inhibited the growth of imatinib-resistant Bcr-Abl-T315I xenografts in nude mice	[101]
Curcumin	In vitro	K562 and LAMA84 cells	↓ miR-21 levels in CML cells -induced PTEN expression in CML cells ↓ AKT phosphorylation and VEGF expression and release ↓ CML cells migration ↓ Bcr-Abl expression in CML cells through the cellular increase of miR-196b -curcumin-treated mice developed smaller tumors	[102]
	In vivo	SCID mice		
Resveratrol	In vitro	K562 cells	-induced apoptosis and phosphorylation of H2AX at Ser139 -stimulated p38 and JNK activation in K562 cells during apoptosis -p38 and JNK regulated resveratrol-induced H2AX phosphorylation in K562 cells ↓ phosphorylation of histone H3 at Ser10	[103]
Resveratrol	In vitro	K562 cells	↓ cell viability and triggered cell apoptosis in K562 cells ↑ Bax/Bcl-2 ratio and release of cytochrome c into the cytosol -induced the activation of caspase-3 ↑ cleaved PARP	[104]
Resveratrol	In vitro	K562 and K562/IMA-3 cells	-inhibited cell growth ↑ in loss of mitochondrial membrane potential ↑ caspase-3 activity -induced apoptosis in K562 and K562/IMA-3 cells	[105]
Phenethyl isothiocyanate (PEITC)	In vitro	K-562, KU812 cells	↑ cytotoxic efficacy of IM PEITC in combination with IM down-regulated the expression of p210^bcr/abl^ in chronic myelogenous leukemia cell lines (K-562) -inhibited the expressions of PKCα, PKCβII, and PKCζ (both phosphorylated and total form) ↓ expression of Raf1 and ERK1/2, two important target proteins in PKC signaling cascade ↓ expression of Raf1 and ERK1/2 through Bcr-Abl and PKC inhibition	[106]
PEITC	In vitro	K562 cells	-induced cell death through the induction of ROS stress and oxidative damage -suppressed cell growth and caused apoptosis by promoting Fas and Fas ligand expression, increasing ROS generation and by the successive release of cytochrome c as well as the activation of caspase-9 and caspase-3	[107]
Indole-3-carbinol	In vitro	K562 cells	-promoted mitochondrial apoptosis of CML-derived K562 cells, as evidenced by the activation of caspases and PARP cleavage ↓ the cellular levels of phospho-Akt and phospho-signal transducer and activator of transcription 5 -activated the p38 mitogen-activated protein kinase ↓ expression of human telomerase and c-Myc	[108]
Emodin	In vitro	K562 cells	-inhibited K562 cell viability in vitro -caused K562 cell morphological changes in vitro -induced K562 cell division cycle arrest at G0/G1 phase in vitro -induced K562 cell apoptosis in vitro and in vivo ↓ Bcl-2 ↑ Bax -induced the activation of caspase-3, -8, and -9 in vitro and in vivo ↓ the tumor volume and tumor weight in nude mice	[109]
	In vivo	BALB/c nude mice		
6-Shogaol	In vitro	K562S and K562R cells	-inhibited cell viability, induced apoptosis in both K562S and K562R ↑ pro-apoptotic Bax gene and ↓ anti-apoptotic BCL-2 gene expression levels significantly in both treated K562S and K562R cells ↑ MDR-1 mRNA expression level in K562S and K562R cells ↓ MRP-1 mRNA expression level in K562S cells	[110]
Parthenolide and DMAPT	In vitro	K562, Meg-01, and KCL-22, HL-60 cells	↓ viability of CML bulk and progenitor cells -induced cell death in CML cells ↑ ROS levels in CML cells -inhibited NF-κB activation in CML cells -inhibited cell proliferation and arrested cell cycle of CML cells in G0 and G2 phases, correlated with down-regulation of cyclin D1 and cyclin A	[111]

**Legend:**↑ increased/up-regulated; ↓ decreased/down-regulated; Bax—Bcl-2-associated X protein; Bcl-2—B-cell lymphoma-2; B-CLL—B-cell chronic lymphocytic leukemia; HSP70—70 kilodalton heat shock proteins; Bcl-xL—B-cell lymphoma-extra-large; FOXM1- Forkhead box protein M1; Gli1—Smoothened and Glioma1; mRNA—messenger ribonucleic acid; Hh—Hedgehog; CDKN1B—cyclin-dependent kinase inhibitor 1B; Akt 1—protein kinase B 1; IFN-γ—interferon-gamma; BTG2—BTG anti-proliferation factor 2; CDKN1A—cyclin-dependent kinase inhibitor 1A; FAS—Fas cell surface death receptor; CFU-Mix—colony-forming unit-mix; BFU-E—burst-forming unit-erythroid; CFU-GM—granulocyte-macrophage colony-forming unit; LTC-IC—long-term culture initiating cell; AIF—apoptosis inducing factor; JAK2—Janus kinase 2; STAT3—signal transducer and activator of transcription 3; AKT—protein kinase B; MAPK—mitogen-activated kinase; JNK—c-Jun N-terminal kinase; ASM—acid sphingomyelinase; cGMP—cyclic guanosine monophosphate; CHES1—checkpoint suppressor 1; PI3K—phosphatidylinositol 3-kinase/protein kinase B; SQSTM1—sequestosome 1; PTEN—tumor suppressor gene phosphatase and tensin homolog; VEGF—vascular endothelial growth factor; miR-196b—microRNA 196b; PARP—poly(ADP-ribose) polymerase; IM—imatinib; PEITC—phenethyl isothiocyanate; Raf-1—proto-oncogene, serine/threonine kinase; ERK1/2—extracellular signal-regulated kinase 1/2; PKC—protein kinase C; ROS—reactive oxygen species; MDR-1—multidrug resistance mutation; MRP-1—multidrug resistance-associated protein 1; NF-κB—nuclear factor-κB; DMAPT—dimethyl amino parthenolide.

**Table 3:** Pharmacological effects of natural antioxidants in acute lymphoblastic leukemia (ALL).

Bioactive Compound	In Vitro/In Vivo/Clinical Study	Cancer Cell Line and Animal Model	Bioactive Effect	References
Quercetin	In vivo	C57BL/6J (CD45.2^+^) and B6.SJL-PtprcaPepcb/BoyJ mice	-enhanced the cytotoxicity of Adriamycin to leukemic cells -improved the survival of mice with T-ALL -enhanced the SOD activity and reduced the MDA content in the heart	[122]
Antho 50	In vitro	Jurkat cells	-induced apoptosis in Jurkat cells ↑ ROS formation ↑ tumor suppressor p73 and cell cycle regulator p21 expression levels -cleaved caspase-3 expression levels ↓ expression levels of p-Akt, survivin, PcG proteins, HDACs, DNMT1, and UHRF1	[117]
Delphinidin-3-*O*-glucoside and delphinidin-3-*O*-rutinoside	In vitro	Jurkat and Molt-4 cell lines	-induced proapoptotic response in Jurkat cells	[121]
DMF	In vitro	YCUB series	-induced G0/G1 cell cycle arrest ↓ the expression of phosphorylated retinoblastoma-associated protein 1 ↑ induced apoptosis in ALL cell lines ↓ the intracellular levels of glutathione -antagonized the cytotoxicity of 4-hydroperoxy-cyclophosphamide, cytarabine, vincristine, and L-asparaginase in all tested ALL cells	[123]
EGCG	In vitro	Jurkat cells	-decreased viability of cells -induced apoptosis of lymphoblastic leukemia cells -enhanced Fas expression in Jurkat cells -increased caspase-3 positive cells	[124]
Curcumin	In vitro	697, REH, RS4;11, and SupB15 cells	-suppressed the viability in B-Pre-ALL cell lines -induced apoptosis in B-Pre-ALL cell lines via activation of caspase-8 and truncation of BID protein ↑ the ratio of Bax/Bcl-2 -induced the dephosphorylation of the constitutive phosphorylated AKT/PKB ↓ the expression of cIAP1, and XIAP ↑ ROS	[125]
Curcumin	In vitro	B6p210 and B6T315I cells	-inhibited proliferation -induced apoptosis ↓ NF-κB levels ↑ p53 levels ↓ c-Abl levels in cells expressing the wild, but not the mutant, BCR-ABL oncogene -improved survival in diseased mice and ↓ WBC and GFP cell counts	[126]
	In vivo	B6 mice		
Resveratrol	In vitro	GC-resistant CEM-C1-15, Jurkat, Molt-4, and GC-sensitive CEM-C7-14 cells	-inhibited the proliferation and induced apoptosis and autophagy in T-ALL cells -induced cell cycle arrest at G0/G1 phase via up regulating CDK inhibitors p21 and p27 and down-regulating cyclin A and cyclin D1 ↓ the expression of antiapoptotic proteins (Mcl-1 and Bcl-2) ↑ the expression of proapoptotic proteins (Bax, Bim, and Bad)	[127]
Pterostilbene	In vitro	Jurkat and Molt-4 cells	↓ cell viability with different extent in two ALL cell lines -induced apoptosis in lymphoblastic cells ↑ Fas expression both in mRNA and surface levels that results in apoptosis signal transduction improvement, which sensitized cells to apoptosis by immune effector cells	[128]
Gambogic acid	In vitro	Jurkat and Molt-4 cells	-inhibited proliferation, induced apoptosis, and activated autophagy in T-ALL cell lines -antileukemic effect against peripheral blood lymphocyte cells in patients with ALL -inhibited phospho-GSK3β S9 protein levels to inactivate Wnt signaling -suppressed β-catenin protein levels	[112]
Gallic acid	In vitro	Jurkat cells	↓ cell viability	[129]
Parthenolide	In vitro	B- and T-ALL cells	-effective against bulk B- and T-ALL cells -prevented engraftment of multiple LIC populations in NOD/LtSz-scld IL-2Rγ^c^-null mice -restoration of normal murine hemopoiesis	[130]
	In vivo	NOD/LtSz-scld IL-2Rγ^c^-null mice		
Thymoquinone	In vitro	Jurkat cells	↓ cell viability of Jurkat cells -induced apoptosis in Jurkat lymphoblastic cell line -combination with doxorubicine lead to a synergistic cytotoxicity	[131]
Thymoquinone	In vitro	CEMss cells	-induced apoptosis in CEMss cells ↑ in chromatin condensation in the cell nucleus ↑ number of cellular DNA breaks in treated cells ↑ apoptosis with cell death-transducing signals by a down-regulation of Bcl-2 and up-regulation of Bax ↑ generation of cellular ROS, HSP70, and activation of caspases -3 and -8 -the mitochondrial apoptosis was associated with the S phase cell cycle arrest	[132]
Indole-3-carbinol	In vitro	NALM-6 cells	-induced cell-growth inhibition, G1 cell-cycle arrest, and apoptosis in NALM-6 cells ↑ the expression of p53, p21, and Bax proteins -induced p53 accumulation and expression of pro-apoptotic p53 target genes ↑ PUMA, NOXA, and Apaf-1 -suppressed NF-κB activation and inhibited the protein expression of NF-κB-regulated antiapoptotic (IAP1, Bcl-xL, Bcl-2, XIAP) and proliferative (c-Myc) gene products -repressed antiapoptotic NF-κB target genes -potentiated doxorubicin-induced apoptosis through caspase activation and PARP cleavage -inhibited doxorubicin-induced NF-κB activation in NALM-6 cells	[133]

**Legend:** ↑ increased/up-regulated; ↓ decreased/down-regulated; T-ALL—T cell acute lymphoblastic leukemia; SOD—superoxide dismutase; MDA—malondialdehyde; ROS—reactive oxygen species; p-Akt—Akt phosphoration; PcG—polycomb group; HDACs—histone deacetylases; DNMT1—DNA methyl transferase 1; UHRF1—ubiquitin like with PHD and ring finger domains 1; DMF- 5,7-dimethoxyflavone; EGCG—epigallocatechin-3-gallate; B-Pre-ALL—B-precursor ALL; Bax—Bcl-2-associated X protein; Bcl-2—B-cell lymphoma-2; Akt—protein kinase B; cIAP1—cellular inhibitor of apoptosis protein-1; XIAP—X-linked inhibitor of apoptosis protein; NF-κB—nuclear factor-κB; c-Abl—Abelson tyrosine kinase; WBC—white blood cell; CEM—human acute T-lymphoblastic leukemia cell line; T-ALL—T-cell acute lymphoblastic leukemia; GFP—green fluorescent protein; cyclin-dependent kinase (CDK); Mcl-1—myeloid cell leukemia 1; Bad—Bcl-2-associated death promoter; LICs—leukemia initiating cells; Hsp70—70 kilodalton heat shock protein; NF-κB—nuclear factor-κB; Apaf-1—apoptotic protease activating factor 1; Bcl-xL—B-cell lymphoma-extra-large; PARP—poly(ADP-ribose) polymerase.

**Table 4:** Pharmacological effects of natural compounds in chronic lymphocytic leukemia (CLL).

Bioactive Compound	In Vitro/In Vivo/Clinical Study	Cancer Cell Line and Animal Model	Bioactive Effect	References
Antho 50	In vitro		-induced apoptosis in B CLL cells -induced an early caspase-3 activation and UHRF1 down-regulation in B CLL cells independently of the status of tumor suppressor genes p53 and p73 ↓ Bcl-2 associated with Bad dephosphorylation -induced PEG-catalase-sensitive formation of ROS in B CLL cells	[144]
Luteolin	In vitro	HG-3 and EHEB cells	-↑ the apoptotic cell population in both CLL cells lines by increasing the activities of caspase-3 and -9 and triggering the intrinsic apoptotic pathway	[145]
Apigenin	In vitro	Eheb cells	-induced apoptosis in human lymphoma B cells in vitro -prevented the reverted mutations	[146]
EGCG	In vitro	CLL B cells	-induced CLL B-cell apoptosis -suppressed Bcl-2, XIAP, and Mcl-1 -down-regulated the phosphorylation of VEGF-R1 and VEGF-R2	[147]
Chrysin	In vitro	CLL and healthy B-lymphocytes	↑ cytotoxicity, intracellular ROS, mitochondrial membrane potential collapse, ADP/ATP ratio, caspase-3 activation and apoptosis -inhibited complex II and ATPases in cancerous mitochondria -promoted apoptosis in CLL B-lymphocytes by selectively targeting of mitochondria	[148]
Chrysin	In vitro	MOLT-4 and JVM-13 cell lines, B-CLL cells derived from 28 patients	-induced the activation of proapoptotic Bax ↓ the expression of antiapoptotic Bcl-2 protein -released cytochrome c from mitochondria into cytosol -activated caspase-3 -induced apoptosis of peripheral blood lymphocytes isolated from human CLL patients	[90]
Resveratrol	In vitro	WSU-CLL and ESKOL cells	-inhibited proliferation in leukemic B-cell lines -induced apoptosis in the two cell lines as well as in B-CLL patients’ cells, as evidenced by the increase in annexin V binding, caspase activation, DNA fragmentation, and decrease of the mitochondrial transmembrane potential -inhibited in situ NO release in WSU-CLL, ESKOL, and B-CLL patients’ cells -down-regulation of the two anti-apoptotic proteins iNOS and Bcl-2	[149]
	In vitro	leukemic lymphocytes from patients with B-CLL		
Resveratrol and quercetin	In vitro	human 232B4 CLL cells	↓ proliferation of human 232B4 CLL cells -induced apoptosis in 232B4 CLL cells through induction of caspase-3 activity -inhibited cell cycle progression -arrested cell cycle mainly in G0/G1	[140]
Curcumin	Clinical study	Twenty-one patients with stage 0/1 CLL	↓ ALC at four patients (20%) ↓ in ALC was accompanied by an ↑ in CD4, CD8, and NK cells	[150]
Curcumin and rapamycin		PBMCs from patients with B-CLL	-induced apoptosis in B-CLL cells obtained from patients with CLL ↑ caspase-9, -3, and -7 activity ↓ anti-apoptotic Bcl-2 levels, ↑ the pro-apoptotic protein Bax	[151]
Allanxanthone C and macluraxanthone	In vivo	xenograft murine model of human CLL	-prolongation of the survival in mice injected with the two xanthones	[152]
PEITC	In vitro	Primary leukemia cells	-killed CLL cells with 17p-deletion -cytotoxic effect against p53-/-leukemia cells from mice in vitro and in vivo ↑ ROS accumulation and GSH depletion in p53-deficient CLL cells ↓ Mcl-1 protein in CLL cells -induced leukemia cell death in mice -prolonged the median survival time of the animals	[153]
	In vivo	TCL1-Tg:p53^+^ mice		
Parthenolide	In vitro	cells isolated from CLL patients	-induced apoptosis in CLL cells -activated the mitochondrial pathway of apoptosis -induced a proapoptotic Bax conformational change, release of mitochondrial cytochrome c, and caspase activation ↓ nuclear levels of the antiapoptotic transcription factor NF-κB and diminished phosphorylation of its negative regulator IκB	[154]
Parthenolide	In vitro	PBMCs from B-CLL patients	-displayed potent cytotoxic and apoptotic effects on B-CLL cells in vitro ↓ in the cell viability of B-CLL cells	[155]
Allicin	In vitro	PBMC cells CD20^+^ cells	-induced in vitro apoptosis -killed the CD20^+^ tumor B cells via apoptosis -exhibited tumoricidal effect in vivo	[156]
	In vivo	BALB/c mice		
Indole-3-carbinol	In vitro	PBMCs cells hMSC-TERT cells	-induced cytotoxicity in CLL cells but not in normal lymphocytes ↓ XIAP and cIAP1/2 and induced caspase 9-dependent apoptosis of CLL cells -sinergic activity with fludarabine in CLL cells and overcame stroma-mediated drug-resistance -mechanism of cell death involved p53-dependent and independent apoptosis -sinergic activity with F-ara-A in all types of CLL cells and restored F-ara-A sensitivity in fludarabine-resistant CLL cells	[157]
	In vivo	C57bl/6 mice		

**Legend:** ↑ increased/up-regulated; ↓ decreased/down-regulated; Antho 50—anthocyanin-rich dietary bilberry extract; B-CLL—B-cell chronic lymphocytic leukemia; UHRF1—ubiquitin like with PHD and ring finger domains 1; Bcl-2—B-cell lymphoma-2; Bad—Bcl-2-associated death promoter; PEG-catalase—membrane permeant analog of catalase; ROS—Reactive oxygen species; XIAP—X-linked inhibitor of apoptosis protein; Mcl-1—myeloid cell leukemia-1; VEGF-R1 and VEGF-R2—VEGF membrane receptors; ADP—adenosine diphosphate; ATP—adenosine triphosphate; NO—nitric oxide; iNOS—inducible nitric oxide synthase; ALC—absolute lymphocyte count; PBMCs—peripheral blood mononuclear cells; GSH—reduced glutathione; NF-κB—nuclear factor-κB; hMSC-TERT—human telomerase reverse transcriptase catalytic subunit; cIAP1—cellular inhibitor of apoptosis protein-1.
